# Supplementary material for: Are carbon emissions trading and green financial instruments synergistic? -Comprehensive quantitative research based on content analysis
Source: PLoS One. 2024 Mar 7;19(3):e0298601. doi: 10.1371/journal.pone.0298601 (PMC10919601; doi:10.1371/journal.pone.0298601)
Supplement: S1 Data — (ZIP) [file pone.0298601.s001.zip › Data/地级市Z_F工作报告.zip!/╜¡╬≈/╥╦┤║╩╨/└┤╫╘╣1⁄2╓┌║┼í╛┬φ┐╦╩2╛▌═°í┐_2019.docx]

各位代表：

现在，我代表市人民政府，向大会报告政府工作，请予审议，并请市政协委员和列席会议的同志提出意见。

一、2018年工作回顾

2018年是宜春加压奋进、砥砺前行的一年。一年来，全市上下深入学习贯彻习近平新时代中国特色社会主义思想和党的十九大精神，认真落实中央和省委、省政府决策部署，在市委的坚强领导下，紧扣建设区域中心城市总目标，凝心聚力谋发展，聚精会神搞建设，全市经济社会实现平稳健康发展，决胜全面建成小康社会取得新的重大进展。

——经济运行稳中向好。全市实现生产总值2180.85亿元、增长8.1%；财政总收入391.4亿元、增长10.4%，其中一般公共预算收入236.42亿元、增长4.9%，税收收入占财政总收入比重84.7%；规模以上工业增加值增长6.3%；500万元以上固定资产投资增长11%；社会消费品零售总额676.03亿元、增长10.7%；实际利用外资8.45亿美元、增长9.45%；城镇新增就业5.81万人，转移农村劳动力8.31万人。县域经济活力迸发，财政总收入超30亿元县（市、区）达到5个。

——重点任务深入推进。“三群两谷一带”产业工程扎实推进，成功实现千亿产业、百亿企业零的突破，中医药全产业链主营业务收入达1006.6亿元，仁和集团主营业务收入达102亿元；锂矿资源整合收储工作基本完成，成功引进合众、九龙SUV等新能源整车项目，锂电产业继续保持快速发展势头。“三个中心、一个基地”加快建设，教体新区总规、城市设计、七大项目红线基本确定，“三校一公园”已立项批复；智慧经济产业特色小镇引进大数据企业46家，呼叫中心、孵化基地、双创基地等重点项目开工建设；宜万经济走廊稳步推进。

——环境质量不断提高。国家生态文明试验区（江西）建设五大改革试点任务取得重大进展。中心城区PM2.5浓度均值同比下降21.6%，全市国考、省考断面主要污染物年均浓度达标率均为100%，环境质量在全省排名靠后的不利局面得到扭转，首次圆满完成生态环保省考核目标。生态文明建设成效明显。

——人民生活持续改善。精准脱贫攻坚有力推进，2.58万贫困人口实现脱贫，59个贫困村摘帽退出。城镇、农村居民人均可支配收入分别为32248元、14975元，分别增长8%和8.9%。年初确定的20项民生实事基本办成，3993个新农村建设点“七改三网”全部完工；6.15万套棚户区改造全面完成；“多险合一”保障平台上线运行；新建25户以上自然村通水泥路1379.7公里。

一年来，面对国际国内错综复杂的环境和挑战，我们按照谋事要实、干事要实、做人要实的“三实”原则，主要做了以下工作。

一是重帮扶、兴实体，产业发展实现新突破。全力实施“产业兴市、工业强市”战略，新出台降成本优环境政策措施55条，帮助企业纾困解难，推动产业加快发展。新型工业化进程加速。全市工业投资增长14.2%。制造业用电量98.9亿千瓦时，拥有省级重点工业产业集群13个，规上工业企业1631家，全国制造业单项冠军示范企业2家，省级“专精特新”中小企业147家、两化融合示范企业28家，贯标认定企业12家，入选江西百强民营企业27家，均居全省前列。新开工建设工业标准厂房267万平方米。省锂电产品质量监督检验中心挂牌运行，丰城高新技术产业园区晋升国家级高新区，宜春经开区获评省级战略性新兴产业聚集区，高安建材产业优化升级列入省级试点。现代农业势头稳健。六大特色农业产业加快发展，中药材种植、富硒农产品基地建设“双百计划”启动实施，中药材种植、富硒农产品基地面积分别达44.6万亩、35万亩；绿色食品原料基地、有机农产品基地、高产油茶林面积分别达191.2万亩、114.8万亩、107万亩；肉牛出栏60万头。“三品一标”认证数达966个，培育省级区域公用品牌2个。发放“财政惠农信贷通”贷款超16亿元。首届中国农民丰收节江西活动在高安成功举办。我市获评全国木本油料（油茶）特色区域示范市、全省耕地保护责任目标考核优秀设区市。完成高标准农田建设54.3万亩，高安、万载、樟树、奉新高标准农田建设荣获全省绩效考评先进。奉新获评“中国好粮油”行动计划示范县，上高列为国家畜禽粪污资源化利用整县推进县，万载入选首批国家农村产业融合发展示范园创建单位，宜丰国家农产品质量安全县创建通过省级验收。樟树现代农业产业园成功申报国家现代农业产业园，丰城富硒园区评为国家农业科技示范园区。现代服务业发展提速。全市接待游客9004.1万人次，实现旅游综合收入805.13亿元，分别增长25.1%、31.7%。成功举办第十二届月亮文化旅游节。“江西宜春·一座四季如春的城市”在中央电视台播放，获得广泛好评。《宜春市温汤地热水资源保护条例》颁布实施，宋城·明月千古情景区建成运营，江西武功山—明月山景区实现门票统一。靖安获评省级全域旅游示范区，高安巴夫洛生态谷获批国家4A级旅游景区，铜鼓乡村旅游扶贫项目入选全国金融支持旅游扶贫重点项目。实现服务业增加值934.17亿元，增长10.9%。金融市场更趋活跃，亚中电子在“新三板”挂牌，光大银行宜春分行揭牌运营，金融机构贷款余额2319亿元、增长21.26%。

二是抓改革、促开放，创新驱动增添新动力。坚持以改革促发展，以开放促改革，创新发展步伐加快。重点改革蹄疾步稳。持续推进“三去一降一补”，关闭煤矿13处、退出产能72万吨。加强国有资源资产整合，市融资担保公司揭牌运营，市本级“1+6”发展平台向实体型企业转化，实现融资401亿元。宅基地管理试点、农村集体产权制度改革稳步实施，绿色殡葬改革有序推进。开放水平持续提升。积极参加赣港会、赣深会、绿发会等重大经贸活动，成功举办2018全球锂电产业链高峰论坛、国际锂电新能源（汽车）产业展览会、大数据产业招商推介会等招商推介活动，引进省外2000万元以上项目322个，袁州海量动力、丰城爱情花卉小镇、上高格林德新能源、宜丰迈丹尼等20个项目投资均超10亿元。宜春—福州、高安—九江铁海联运快速班列开通运行，国际贸易实现“单一窗口”全覆盖。明月山机场通航北京、天津、上海、广州、深圳、重庆等16座热点城市，旅客吞吐量达63.98万人次。创新活力有效释放。预计全社会研发经费投入占GDP比重超1.25%。新增国家高新技术企业187家，国家科技创新创业人才1名，院士工作站4个，省级创新平台载体23家、重大科技研发专项2项、科技协同创新体3个。大众创业万众创新蓬勃开展，新增市场主体6.7万户。中心城区416套人才公寓分批投入使用。

三是打基础、谋长远，城市建设展现新风貌。突出规划引领，建管并重，“里子”“面子”同步升级。空间布局更加优化。谋划构建“五组团、双十字、一环城”中心城市新格局，完成了教体新区可行性研究论证报告、宜阳新区（大塘片区）及袁州新城、古城核心区城市设计和宜万经济走廊规划编制工作。争取到中心城区环城高速与宜春至吉安遂川高速公路同步实施，建设宜春“十字”型高铁枢纽写入2019年省《政府工作报告》，中心城区“双十字、一环城”高速交通路网取得实质性进展。城市功能更加完善。实施中心城区各类民生项目135个，累计完成投资196.5亿元。明月山机场站坪扩建、四方井水利枢纽工程、220国道绕城改建、宜春大道改造有序实施。城市管理更加精细。深化综合行政执法体制改革试点，城市执法和管理实现重心下移、力量下沉、事权下放。拆除城市建成区违法建设40.87万平方米，环城西路、建材市场、贸易广场等重点区域环境综合整治取得明显成效。宜春成功入选全国文明城市提名城市。

四是强治理、守底线，生态文明凸显新亮点。保持加强生态文明建设的战略定力，守住绿水青山底色。污染治理力度加大。主动策应长江经济带“共抓大保护”攻坚行动，认真做好中央、省环保督察和生态环境部约谈反馈问题、交办信访件整改工作。深入开展大气污染防治夏季和冬春季攻坚行动，着力控煤、减排、管车、降尘、禁烧、治油烟。大力实施中心城区生活污水分段式截污处理工程，新改建工业园区污水配套管网133.1公里，信息化一体监控平台实现工业园区全覆盖。试点工作进展顺利。扎实推进生活垃圾分类和减量工作，试点范围覆盖中心城区63.2%的人口，“生态+大健康”产业发展规划、2013—2016年度自然资源资产负债表编制完成，靖安农村环境整治政府购买服务试点工作进展顺利，高安自然资源统一确权登记通过自然资源部评估验收。生态建设亮点纷呈。大力推进秀美乡村建设，建成示范线11条、示范村庄74个。推行林长制、河（湖）长制，生态资源管护机制更加有力。奉新获评省级生态县，靖安“一产利用生态，二产服从生态，三产保护生态”的做法，得到习近平总书记点赞。

五是补短板、办实事，民生保障再上新台阶。积极回应群众诉求，着力保障改善民生，人民群众拥有更多获得感、幸福感和安全感。脱贫攻坚成效明显。大力实施脱贫攻坚春季攻势、夏季整改、秋冬会战三大行动，深入推进十大扶贫工程，全市贫困发生率由1.42%下降至0.86%。扎实推进城镇贫困群众脱贫解困，摸底认定工作基本完成。民生保障坚实有力。城镇登记失业率为3.03%。城乡居民社会保障水平稳步提高。全市新建、改扩建城区学校31所，新增学位4.18万个，中心城区建成学校7所、新增学位1.34万个。教育教学质量稳步提升，全市高考二本以上上线人数约占全省14%。宜春学院在教育部本科教学工作审核评估中获评区域特色鲜明、优势突出、极具发展潜力的地方应用型大学，南昌理工学院丰城校区开工建设。奉新成功创建全国中小学校责任督学挂牌督导示范县。我市获评全国基层中医药工作先进单位。新改建基层综合性文化服务中心1072个，省七〇三电视台新台开播，宜春美术馆开馆。新增“中国好人”19人。成功举办中国·宜春国际农耕健身邀请赛暨第七届全国农耕健身大赛、第25届全国冬泳锦标赛、首届宜春杯赣湘鄂边区机关干部篮球邀请赛。社会大局和谐稳定。深入开展十大专项整治，安全生产形势保持平稳。连续十年实现地质灾害防治“零伤亡”。积极化解矛盾纠纷，民情家访日记工作获评新时代“枫桥经验”江西实践优秀成果。扎实推进扫黑除恶专项斗争，打掉涉黑涉恶团伙71个，抓获犯罪嫌疑人1967人。社会环境安定有序，公众安全感指数和群众满意度保持在96%以上。

六是转作风、优服务，自身建设取得新成效。扎实推进“五型”政府建设，坚决整治“怕、慢、假、庸、散”等作风顽疾，着力转变作风、提升效能。政务服务持续优化。市行政审批局正式运行，实现“一枚印章管审批”。深入推进“一次办好、一次不跑”改革，精简和调整市本级权力事项502项，不动产“登记+交易”一体化改革获国务院通报表扬。市民服务中心投入使用。“12345”政府服务热线平台平稳运行，来电接通率达97.14%，服务对象满意率达88.57%。依法治市扎实推进。开展了规范性文件审查备案清理，取消调整各类证明事项153项。依法接受市人大及其常委会的法律监督、工作监督和市政协的民主监督，人大代表建议、政协提案办复率、满意率和基本满意率均达100%。廉政建设引向深入。严格执行中央八项规定及其实施细则精神，持之以恒纠“四风”，力戒形式主义、官僚主义，“三公”经费进一步下降。坚决全面彻底肃清苏荣案余毒，认真履行“一岗双责”，强化审计监督，严肃查办了一批违纪违法案件，反腐败斗争取得扎实成效。

与此同时，国防动员和后备力量建设、人民防空、拥军优属、国家安全、民族宗教、外事侨务、妇女儿童、青少年等工作继续加强；邮政通信、石油、烟草、气象、水文、盐业、无线电管理、防震减灾、统计、档案、史志、残疾人、红十字会和社会科学等工作取得新成效。

各位代表！

过去一年取得的成绩，是习近平新时代中国特色社会主义思想科学指引的结果，是省委、省政府和市委正确领导的结果，是市人大、市政协监督与支持的结果，是全市人民团结奋斗的结果。这也再次表明，宜春人民有战胜任何艰难险阻的勇气、智慧和力量，宜春没有做不好的事情。在此，我代表市人民政府，向全市广大干部群众，向人大代表、政协委员，向各民主党派、工商联、无党派人士和人民团体，向驻宜部队官兵以及中央、省驻宜单位，向离退休老同志，向所有关心支持宜春发展的各界朋友，表示衷心的感谢和崇高的敬意!

需要向代表说明的是，受国内外经济下行压力加大和我市主动实施以发展增量挤压历史水分，以及国家统计局执法检查等因素影响，GDP增速低于全省0.6个百分点，低于预期目标0.7个百分点；规上工业增加值增速低于全省2.6个百分点，低于预期目标2.7个百分点。同时，我们更加清醒地认识到，全市经济社会发展还面临不少困难和问题。比如：经济总量不大、质量不高，产业特色不明显，支柱产业不够强，创新能力还需增强，能够支撑宜春经济发展特别是中心城区“三个中心、一个基地”大梁的大产业、大项目缺乏；营商环境与市场主体、企业家的期待还有较大差距，行政服务效率还不高；城市建设与区域中心城市定位不相适应，尤其是中心城区实力不强、老城区和城郊出城道路环境比较乱，“城市病”还不同程度存在；环境保护、安全生产压力大、任务重；就业、教育、医疗、养老等民生保障领域还有不少短板，老百姓的一些合理诉求没有很好地解决；政府工作还存在不足，一些改革发展举措落实不到位，“怕、慢、假、庸、散”等作风顽疾尚未全面根治，少数干部不作为、慢作为甚至乱作为的现象仍然存在。我们一定直面问题和挑战，勇于担当，恪尽职守，竭尽全力做好各项工作，绝不辜负全市人民的期待！

二、2019年主要工作

今年是新中国成立70周年，是决胜全面建成小康社会、实现第一个百年奋斗目标的关键之年，是建设区域中心城市的攻坚之年，做好全年工作意义重大。新的一年，尽管我国经济发展面临的环境更复杂更严峻，可以预料和难以预料的风险挑战更多更大，但长期向好的基本面没有变，我国发展仍处于重要战略机遇期。以习近平同志为核心的党中央洞察国际大势，把准时代脉搏，在经济、政治、文化、社会民生、生态文明等领域打出一系列“组合拳”，为中国经济注入了强心剂。省委十四届六次全会着眼推动江西高质量跨越式发展，提出了“创新引领、改革攻坚、开放提升、绿色崛起、担当实干、兴赣富民”工作方针和“六大突破、三大提升”工作部署，使我们的发展取向、工作导向、奋斗指向更加明确、更加集中。特别是省委十四届七次全会强调“宜春要大力发展锂电新能源、中医药、大健康等绿色产业，联动新余、萍乡一体发展，加快建设区域中心城市”，凸显了宜春在江西西部地区的中心地位，赋予了宜春新的历史使命。我们要进一步提振干事创业的精气神，坚定不移把宜春的事情办好、办实、办精彩，展现新时代宜春人民的新担当、新作为、新风貌。

今年政府工作的总体要求是：高举习近平新时代中国特色社会主义思想伟大旗帜，全面贯彻党的十九大、十九届二中、三中全会和中央经济工作会议精神，从更高层次贯彻落实习近平总书记对江西工作的重要要求，按照省委十四届六次、七次全会部署，坚决落实省委“创新引领、改革攻坚、开放提升、绿色崛起、担当实干、兴赣富民”工作方针，坚持稳中求进工作总基调，坚持新发展理念，坚持高质量跨越式发展首要战略，坚持以供给侧结构性改革为主线，坚持以区域中心城市建设统领全市经济社会发展工作，坚定不移实施“产业兴市、工业强市”战略，坚定不移打好三大攻坚战，坚定不移深化改革开放，坚定不移保障和改善民生，坚定不移推进“五型”政府建设，保持经济持续健康发展和社会大局稳定，为全面建成小康社会收官打下决定性基础，以优异成绩庆祝新中国成立70周年。

今年全市经济社会发展主要预期目标是：生产总值增长8.1%左右；财政总收入增长6%左右，其中一般公共预算收入增长5%左右；规模以上工业增加值增长8.3%左右；500万元以上固定资产投资增长8.8%左右；社会消费品零售总额增长10.5%左右；外贸出口稳中提质，实际利用外资增长6%左右；城镇居民人均可支配收入增长7.5%左右；农村居民人均可支配收入增长8%左右；居民消费价格总水平涨幅控制在3%以内；城镇登记失业率控制在4.5%以内；节能减排完成省下达的计划任务。实现这些目标，需要付出艰苦努力，在实际工作中，要跳起来摘桃子，争取更好结果。

具体工作中要把握好以下原则：

旗帜鲜明讲政治，坚持把对标对表抓落实作为根本要求。讲政治是具体的不是抽象的，是无条件的不是有条件的，必须自觉对标对表中央、省委省政府和市委决策部署，做到任何时候都坚持党对经济工作的领导，任何事情都从讲政治的高度去审视、谋划和推进，始终保持政府工作的正确方向。

矢志不移抓发展，坚持把做大总量、提高质量作为头等任务。发展是解决宜春一切问题的基础和关键，必须坚定不移紧紧扭住经济建设这个中心不动摇、不松劲、不懈怠，牢牢把握发展主动权，着力在项目建设、招商引资、科技创新、重点改革、扩大开放、区域协调、城乡融合、厚植人文等方面精准发力、持续用力，进一步做大做强做优经济总量。

全力以赴强实体，坚持把主攻工业作为强市之基。实体经济是宜春走向未来的根基，必须坚持主攻新型工业化不动摇，着力改造提升传统产业、发展壮大高新技术产业和战略性新兴产业，挺起宜春高质量跨越式发展的脊梁。

千方百计扩投资，坚持把项目建设作为重中之重。项目建设是稳增长的“压舱石”，重大项目建设对我市经济社会发展具有关键性、引领性、支撑性作用，必须树立“不抓项目是失职、不会抓项目是不称职”鲜明导向，加快推进重大项目的谋划包装、申报争取、落地建设等工作，以项目建设稳投资、提质量、增后劲。

齐心协力优环境，坚持把服务企业发展作为光荣使命。非公经济贡献了约宜春60%的GDP，85%以上的固定资产投资和税收收入，必须强化“以主动服务企业发展为荣、以阻碍发展为耻”的观念，着力优化营商环境，激励和引导广大干部主动服务企业，真心实意帮助企业，把市场主体的活跃度保持住、提上去，稳定企业家信心，这是稳增长的关键所在。要坚定不移支持民营企业改革发展，让民营经济和其他各类经济在宜春有更加广阔的舞台。

尽心尽力惠民生，坚持把满足宜春人民对美好生活的需求作为奋斗目标。保障和改善民生没有终点站，必须践行以人民为中心的发展思想，把人民群众的小事当作自己的大事，解决好老百姓最关心最迫切的问题，努力让宜春人民的生活一天更比一天好。

实现上述目标要求，我们要突出重点、把握关键，扎实做好以下几个方面工作。

（一）擂响工业强市“进军鼓”，加快构建现代产业体系。以更大的力度、更强的决心、更实的举措发展新型工业，优服务、育龙头、聚集群，推动工业经济量质齐升，重塑宜春制造业辉煌。

培育壮大产业集群。紧扣“四年翻一番、决战工业八千亿”目标，聚力建设“三群两谷一带”产业工程。开展“机器换人”行动助推装备制造业焕发新生机，对接京九（江西）电子信息产业带，科学布局电子信息产业，力争机电产业集群主营业务收入增长15%。实施“建材产业优化升级工程”，支持优势陶瓷企业兼并重组做大做强，培育发展各类新型墙材建材，力争建材产业集群主营业务收入增长6%。开展纺织鞋革增品种、提品质、创品牌“三品”行动，支持企业进行智能化改造，力争纺织鞋革产业集群主营业务收入增长6%。围绕建设“宜春锂谷”，提升锂矿资源氧化锂综合提取利用率，主攻锂电池正极材料、动力和储能电池等中端环节，加快推进整车项目建设，力争锂电产业主营业务收入增长30%以上，谋划建设赣西锂电新能源产业带。围绕建设“宜春药谷”，全力打造“中医药·世界看中国，中国看宜春、看樟树”品牌，大力实施樟树“中国药都”振兴工程和袁州“一谷一城一港”计划，抓好樟树岐黄小镇建设，扩大中药材种植规模，提升医药制造水平，推进医养结合，坚决捍卫“中国药都”正统地位，力争中医药全产业链主营业务收入超1100亿元。发展粮食、畜禽、油茶等农副产品精深加工，推进绿色有机食品规模化经营，力争绿色食品产业主营业务收入增长5%。

着力完善园区功能。开展集群式项目“满园扩园”、“两型三化”园区管理提标提档行动，推动工业园区向现代化产业园区转型升级，力争全市工业园区主营业务收入增长9%左右。完善宜春智慧工业平台，实现规上工业企业、园区企业全覆盖。坚持集约节约用地，加强闲置土地整治及开发，盘活“僵尸企业”，力争园区亩均投资强度、主营业务收入均突破400万元，亩均税收突破25万元。积极探索工业“标准地”建设，抓好500万平方米标准厂房建设，力争国家级开发区至少引进1个投资50亿元以上项目，省级工业园区至少引进1个投资20亿元以上项目。加快推进赣湘边区经贸合作产业园建设，致力打造“五区一堡一极”。支持宜春经开区裂变式发展，完善功能配套，推进产城融合，推动主导产业集群集聚，力争主营业务收入达330亿元。

大力发展民营经济。把稳定企业家特别是民营企业家的信心放在首位，毫不动摇地支持保护民营经济发展，全面落实各项惠企便企政策，让企业家放心放胆创业，让民营企业成长为“参天大树”。壮大宜春制造业50强，培育“专精特新”和“小巨人”企业，推动200家规上工业企业实施技术改造，力争更新优化“专精特新”企业40户、新增“小巨人”企业5户。感情上要像对待兄弟姐妹一样对待企业家，像尊重自己的老师一样尊重老总，像尊重科学家一样尊重企业家，敢“亲”真“清”，既坦荡真诚地同民营企业家交朋友，又满腔热情地为企业服务。落实企业上市“映山红行动”，力争新增境内外上市企业2家。设立非公企业维权中心，开展“走亲连心”活动，全力帮助企业排忧解难。建立政银企互联互通机制，规范发展企业转贷基金，发挥融资性担保机构对企业的增信作用，引导更多的金融活水加速流向实体经济、浇灌民营企业。

（二）打好项目建设“攻坚战”，切实增强经济发展后劲。全面实施“项目攻坚年”行动，重点推进410个大中型项目，总投资2960亿元，完成年度投资960亿元，在全市掀起抓项目建设、扩有效投资的新高潮。

精心策划谋项目。围绕主攻新型工业化、实施乡村振兴战略和发展现代农业、发展现代服务业、提高基础设施保障能力、发展绿色生态经济和环保产业、发展社会事业，储备和争取一批重大项目。抢抓国家保持基础设施领域补短板力度和全省五大领域基础设施建设“补短板”三年攻坚行动计划的重大机遇，做好项目谋划、储备申报工作，力争更多项目进入国家和省重大项目“笼子”。全面梳理“十三五”规划中具备启动条件的重大项目，抓紧开展前期工作，争取尽早开工。把握政策“窗口期”，争取中央预算内投资、企业债发行等各类资金支持。

加大力度上项目。加强项目建设管理，实行挂图作战，责任明确到人，时间细化到月，确保达到进度要求。产业项目方面，加快推进宜春经开区合众汽车、袁州万宝科技、万载瑛维箱包等项目建设，争取丰城唯美二期、奉新云威新材料、高安通瑞新能源、宜丰永兴特钢新能源等项目竣工投产。重大基础设施方面，做好铜鼓至通城高速公路、武宁经靖安、奉新、高安、樟树至兴国高速公路等项目前期工作，加快推进蒙华煤运通道、昌吉赣客专宜春段等项目建设。能源水利方面，做好宜春港、靖安洪屏抽水蓄能电站二期、奉新抽水蓄能电站等项目前期工作，加快推进四方井水利枢纽工程、龙头山水电站枢纽工程建设。

优化服务推项目。完善跟踪推进、协调服务、调度督查机制，提高项目落地率、开工率、竣工率和达产率。落实领导服务企业、挂点企业、帮扶企业制度，经常性深入企业听取诉求，了解生产经营状况，及时协调解决企业生产经营遇到的困难和问题。进一步压缩企业开办时间和工程建设审批时间，优化市场准入和投资建设环境。开展项目建设环境集中整治，对违纪违法者“铁腕整治，绝不留情”，对遵纪守法者“无事不扰，有求必应”。

（三）做实区域协调“大文章”，奋力建设区域中心城市。着力提高中心城区辐射带动能力，推进区域融合发展，重点实施360个城建项目，总投资837亿元，实现城乡一体、共同繁荣。

做强综合实力，提高中心城区首位度。按照“精心规划、精致建设、精细管理、精美呈现”的要求，增强城市空间统筹能力、人文魅力、宜居能力、治理能力，加快建设“三个中心、一个基地”，构建完善“五组团、双十字、一环城”的中心城市新格局。行政中心组团方面，启动第二商业中心和新市民中心建设，抓好智慧经济产业特色小镇重点项目建设，完成“云上宜春”大数据平台建设，引进大数据产业项目100个以上。袁州古城组团方面，实施文化复兴工程，打造王子巷—大坪里历史文化街区、沙子巷—五眼井文化街区、宜春台历史文化公园、灵泉池遗址公园，推进状元洲、大成殿提升和文笔峰重建。明月山景区组团方面，大力发展全域全季旅游，加快建设中科院院士科学家康养基地、仁和矿泉水厂、洪江禅意小镇等重大项目。教体新区组团方面，扎实推进“三校一公园”建设，着力引进一批能够快速集聚人气、快速建设的优质项目。宜万经济走廊组团方面，适时启动万载“撤县设区”，以宜万同城快速通道的建设推动宜上万融合发展。抓紧谋划武宜深高铁项目，开工建设环城高速、宜春至吉安遂川高速公路，加快推进宜春大道改造，通过建设“双十字、一环城”及相匹配的快速通道，把中心城区打造成为全省区域性综合交通中心。

强化“宜春意识”，增强宜春人民认同感归属感。深化全国文明城市创建，大力实施城市功能与品质提升三年行动，以改促建上档次、以管促优出精品、以文铸魂提品位，加快建设大美、灵气、秀丽宜春。深入开展公共环境整治、公共秩序严管、公共服务优化、公益广告提质、公民素质提升、入户宣传“访万家”六大行动，完善城市基础设施配套，改造背街小巷、农贸市场、老旧小区，进一步提升城市宜居品质。扎实推进文化强市，全面唱响“袁州文化”品牌，把袁州古城元素和状元文化元素融入城市建筑、历史文化街区、旅游观光景点，坚定宜春文化自信，凝聚宜春文化共识。高标准、高质量、高效率筹办好2019年全省旅发大会，抓好56个重点旅游项目建设，争取每个县再创1个国家4A级旅游景区，提升全市文旅产业发展水平。规划建设文化创意产业园，促进宜春·国际儿童动漫小镇落地，支持靖安三爪仑旅游区创新中国成立家5A级旅游景区，铜鼓汤里温泉、高安巴夫洛创新中国成立家旅游度假区。抓好万载田下古城、宜丰天宝古城、高安贾家古村、丰城白马寨等历史街区和传统村落的整体保护。突出产城融合，大力发展生活性和生产性服务业，培育彰显城市特色的产业体系。推进城市管理立法工作，加快智慧城市建设，提高城市治理智能化、精细化水平。加大违法建设治理力度，全面开展生活垃圾分类和减量工作，切实加强城乡环境综合整治，突出抓好丰城、樟树昌吉赣高铁沿线村庄整治和规划管控，让人文底蕴流光溢彩，让文明行为成为习惯，让人居环境更加美好。

深入挖掘潜力，打造县域经济“升级版”。鼓励丰樟高、奉新、靖安深度融入大南昌都市圈，以做大做强中心城区拉动万载、上高、宜丰、铜鼓等地发展，推进丰樟高创新协同发展示范区、靖奉宜铜“绿水青山就是金山银山”实践创新示范区建设。支持丰樟高主动融入大南昌都市圈产业链，参与分工配套，加强基础设施互联互通，实现“借道超车”；支持奉新、靖安发展生态旅游、休闲农业、绿色康养等现代服务业，打造南昌后花园；支持袁州、万载、上高同城发展，同步推进先进制造业小镇、物流小镇建设和“生态+大健康”产业发展，培育高质量发展新增长极；支持宜丰、铜鼓发展现代农业、生态工业、旅游业，打通绿水青山向金山银山转换的通道，进一步激发县域经济活力，形成竞相比拼、赶超发展的新局面。

（四）下好改革开放“先手棋”，持续释放发展动力活力。在改革攻坚中找办法、在扩大开放中求突破，走好创新发展之路，不断激发市场活力和社会创造力。

推进改革攻坚。认真贯彻中央“巩固、增强、提升、畅通”八字方针，持续深化供给侧结构性改革。全面完成机构改革任务，继续深化事业单位改革。构建完善“1+6+10”发展平台体系，增强发展平台市场运作能力。完善农村承包地“三权”分置制度，推进农村集体产权制度改革，实施经营性资产股份合作制改革试点，力争两年内完成宅基地管理试点工作。抓好公立医院改革，开展市直公立医院人员总量管理试点。调整中医医疗服务价格。积极稳妥推进绿色殡葬改革。

构建双向开放格局。深度融入“一带一路”、长江经济带和赣江新区等国家战略，打造内陆双向开放新高地。坚持把招商引资作为第一要事，以“洪荒之力”抓招商引资，健全新型的开放型经济考核奖励办法，造浓全民招商氛围，力争引进亿元以上项目200个。紧扣产业发展需求，对世界500强、国内500强、行业龙头企业、知名民企进行精准对接，着力引进一批延链、补链、强链、壮链型项目。瞄准粤港澳大湾区、闽东南、江浙沪、京津冀、武汉都市圈和长株潭城市群等重点区域，组织专业小分队开展驻点招商。办好第二届全球锂电产业链高峰论坛、宜春重点产业（赣湘鄂）招商推介会、首届中医药博览会暨第50届全国药交会、第十三届月亮文化旅游节。发动异地宜春商会、行业协会、工商联、企业家队伍等社会力量参与招商，开展“三请三回”和“三企”入宜工作，形成引进一个、带动一片的效果。加快口岸功能区建设，提升高安铁路专用线货运量，发挥万载烟花爆竹监装功能，推进樟树河东港建设，启动袁州保税物流中心（B型）项目申报工作。畅通开放物流通道，促成宜春—宁波港快速班列尽早开通。设立市级外经发展专项资金，鼓励农业、矿业、基础建筑等行业重点企业开展境外投资。

突出创新引领。大力实施创新驱动“5511”工程倍增计划和高新产业引培工程，全面推进产业创新、企业创新、产品创新、市场创新，力争全社会研发经费投入占GDP比重达1.4%，新增省级高新技术产业园1—2家、国家高新技术企业100家，获批国家级众创空间和星创天地2个、省级创新平台载体10个。加快科技成果转移转化，抓好 “03专项”试点示范拓展区建设。加强知识产权创造、保护和应用，力争新增中国驰名商标1—2件、地理标志保护产品1—2件、专利申请量7200件，每万人发明专利拥有量达2件。实施人才强市战略，重点引进高水平创新创业团队、高层次创新创业人才，力争新增省级以上创新人才和团队10个，院士工作站3个。

（五）开创乡村振兴“新天地”，推动宜春由传统农业大市向现代农业强市转变。按照“产业兴旺、生态宜居、乡风文明、治理有效、生活富裕”的总要求，加快农业农村现代化，建设农业强、农村美、农民富的新乡村。

促进农业全面升级。坚持质量兴农、绿色兴农、品牌强农，调优产业结构、调绿生产方式、调顺经营体系。落实粮食安全责任制，发展粮食产业经济，新增建设高标准农田49万亩。深入开展“大棚房”问题专项清理整治行动，坚决遏制农地非农化现象。壮大六大特色农业产业，加快实施中药材种植、富硒农产品基地建设“双百计划”，确保中药材种植、富硒农产品基地面积分别新增30万亩、20万亩以上；绿色食品原料基地、有机农产品基地、高产油茶林面积分别达到240万亩、150万亩、114.7万亩，肉牛出栏70万头。推进休闲农业“两圈一廊”建设，争创省级田园综合体2个。强化农产品质量安全监管，创新中国成立家农产品质量安全市。聚力打造农产品品牌，培育壮大宜春大米、宜春茶油等一批区域性公共品牌，力争新增省级绿色有机农产品示范县1个，“三品一标”认证产品突破1000个。推广“123+N”智慧农业建设，做大“供销e家”等电商平台。

加快农村全面进步。编制完善县域村庄空间布局和整治建设规划，完成自然村组整治建设2500个。统筹推进秀美乡村建设“1+6”工作，每个县（市、区）再创1—2条示范线，每个乡镇建设1个特色示范点。加快推进“四好农村公路”建设，方便基层群众出行。力争发放“财政惠农信贷通”贷款15亿元以上，扩大农业政策性保险覆盖面。大力发展村集体经济，确保消灭30%的“空壳村”，新增30%集体收入超5万元的村。

推动农民全面发展。培育发展农业新型经营主体，通过推广适度规模经营提高农业生产效率、增强农民技能素养、促进农民增收致富，力争市级以上农业产业化龙头企业达445家、注册农民合作社达7500家、家庭农场达2500家。加强“惠农服务中心”示范点建设，进一步优化农村基层服务组织体系。大力实施新型职业农民培育工程和“一村一名大学生工程”，培养一批扎根农村、发展农业的新型农民和农村实用人才。

（六）唱响绿色崛起“主旋律”，打造生态文明建设“宜春样板”。积极探索以生态优先、绿色发展为导向的高质量发展新路子，加大生态系统保护力度，发展绿色产业，提升环境质量，努力将美丽生态演绎成“美丽经济”。

发展绿色经济。落实国家生态文明试验区（江西）建设试点任务，加快发展“生态+大健康”产业，建设医疗健康养生区、医药健康食品集聚区和生态休闲康养区，打造一批重点示范工程。开展工业绿色发展专项行动，推进园区循环化改造，支持丰城、樟树建设国家循环经济示范县，宜丰、铜鼓、奉新创新中国成立家级生态示范县。做好重点用能单位节能减排降碳工作，力争万元GDP能耗下降3%，推动绿色低碳生活方式成为全社会的广泛共识、自觉行动。

加强环境治理。抓好中央、省环保督察和生态环境部约谈问题整改工作，推动环境质量持续改善。深入推进蓝天保卫战，加强大气污染源管控，强化大气污染专项治理，确保中心城区PM2.5浓度均值控制在省考核目标内，县（市）环境空气质量在全省实现进位。打好碧水保卫战，推进工业园区和城镇污水处理厂提标改造，抓好乡镇集中式饮用水水源地排查整改工作，加大重点流域综合治理力度，确保国控、省控、县界断面水质全部达到III类及以上。实施土壤污染防治行动计划，加强畜禽养殖污染防治，开展化肥农药零增长行动，严控重金属污染物排放总量。

筑牢生态屏障。完成“三线一单”编制，开展生态保护红线勘界落地试点。加强山水林田湖草综合治理，推进湿地保护与修复、水土流失综合整治、矿山环境治理和修复等绿色生态工程建设。抓好风景名胜区和高速铁路、高速公路沿线等重点区域森林“四化”建设，支持奉新、铜鼓创新中国成立家森林城市，力争全市完成人工造林10.68万亩，封山育林14.39万亩，退化林修复19.11万亩，森林抚育46.9万亩。开展自然保护地大检查和自然保护区监督检查专项行动，及时查处纠正违法违规行为。

完善监管体系。整合生态环境执法资源，组建生态环境保护综合执法队伍。完善空气、水、土壤、生态、污染源生态环境监测网络，做好生态环境质量预警预测工作。探索建立流域生态补偿、生态环境损害赔偿等制度，以奖代补、以奖促治，实现生态环境长效治理。

（七）织牢民生福祉“保障网”，提高人民群众生活质量。更加注重在发展中补齐民生短板、托住民生底线，筹集财政性资金460亿元，集中力量办好26件民生实事，进一步增强全市人民的获得感、幸福感、安全感。

坚决打赢脱贫攻坚战。深入推进精准帮扶“十大行动”，精准落实产业扶贫、就业扶贫、教育扶贫等措施，确保高质量完成脱贫任务。坚持资金、项目、政策优先配置，完善贫困村交通、饮水安全、水利工程、网络和文化服务设施。开展“百企帮百村” 行动，推广“爱心超市，积分激励”正向激励模式，实现扶贫与扶智、扶志并举。深入推进扶贫领域腐败和作风问题专项治理，坚决整治脱贫攻坚中的形式主义、官僚主义，坚决杜绝“数字脱贫”“虚假脱贫”。

关心关切困难群众生活。全面落实基本生活保障、就业创业、医疗保障、教育救助、社会保险、住房安全保障、临时救助、社会帮扶等政策，统筹推进城镇贫困群众脱贫解困工作。拓宽就业渠道，力争新增城镇就业4.72万人，转移农村劳动力6.67万人。提高养老待遇、高龄长寿津贴标准。完善农村留守儿童、妇女、老年人关爱服务体系。扩大住房和城乡建设领域有效投资和消费，抓好棚户区改造和“三限房”建设，满足群众多样化住房需求。

繁荣发展社会事业。坚持教育优先发展战略，发展普惠性学前教育，解决义务教育大班额问题，普及普通高中教育，提升职业教育办学内涵，进一步提高普通高等教育办学质量和办学层次，让更多的学子享受优质教育资源。谋划推进市老年人学习、活动场所建设。实施教师教育振兴行动计划，让尊师重教在宜春蔚然成风。深入推进国家健康城市试点，做好国家卫生城市复审迎检，加快市人民医院北院二期、市本级中医院建设。办好系列农耕健身大赛，抓好新水上运动基地一期工程建设。繁荣发展哲学社会科学、文学艺术和档案事业。建设融媒体中心，推进中心城区新时代文明实践中心点建设。新改扩建基层综合性文化服务中心418个，进一步完善基层公共服务网络。

营造和谐稳定环境。压紧压实安全生产责任，加强安全生产隐患排查治理，深化安全生产十五大专项整治，有效防范和遏制各类安全事故发生。抓好应急管理综合平台项目建设，强化气象、地震、地质灾害等预警监测。推进质量强市工作，保障食品药品安全。严厉打击非法集资和金融乱象，坚决守住不发生区域性金融风险的底线。深入开展扫黑除恶专项斗争，着力铲除黑恶势力滋生土壤。始终保持打击各类违法犯罪的高压态势，构建覆盖全社会立体化社会治安防控体系，坚决处置涉众型、风险型经济案件。推广“互联网+民情信息”工作模式，妥善解决群众合理合法诉求。加强国防动员和后备力量建设，深化军民融合，做好人民防空和优抚安置双拥等工作，关爱退役军人，推进各级退役军人服务保障体系建设。

三、加强自身建设，打造人民满意政府

新时代呼唤新作为。我们要倍加珍惜和维护全市业已形成的干事创业的良好氛围、稳中向好的发展态势、风清气正的政治生态，以“五型”政府建设为总抓手，以谋事要实、干事要实、做人要实“三实”为工作要求，不断提升理解力、执行力、操作力、创造力，当好新时代的答卷人，更好满足人民对美好生活的新期待。

（一）坚定立场，建设忠诚型政府。各级政府要树牢“四个意识”、坚定“四个自信”，坚决做到“两个维护”，自觉在思想上政治上行动上同以习近平同志为核心的党中央保持高度一致。深入挖掘宜春厚重的红色历史和纯正的红色基因，不忘初心、牢记使命，把对党和人民事业的绝对忠诚铸入政府工作人员的血脉和灵魂，贯穿和体现到政府工作的全过程、各方面。

（二）大胆实践，建设创新型政府。坚持将改革开放进行到底，聚焦聚力推动宜春高质量跨越式发展，筹划一批有影响力的重大活动，谋划一批打基础、增后劲、管长远的重大项目，着力破解制约发展的突出瓶颈和深层次问题。担负起创新型城市建设的政府之责，精心搭建创新平台、集聚创新人才，让人才创业有机会、干事有平台、发展有空间，形成集聚人才的强大“磁场”。

（三）敢于负责，建设担当型政府。进一步树立“聚焦发展、崇尚实干”的工作理念，倡导“事事马上办、人人钉钉子、个个敢担当”的精神，培育“狮子型”干部，锻造“狮子本领”。健全完善容错纠错机制，旗帜鲜明为敢于担当、踏实做事、不谋私利者撑腰鼓劲，用制度保障解除广大干部的后顾之忧，激励和调动广大干部的干事创业激情。坚持底线思维，增强忧患意识，提高防范和化解风险的能力，确保宜春经济持续健康发展和社会大局稳定。

（四）提升效能，建设服务型政府。坚持把服务作为政府的天职，重塑政府和市场的关系，提升政府服务效能。各级政府要强化艰苦奋斗、勤俭节约的意识，以政府过紧日子换取企业和群众过好日子。进一步深化“放管服”改革，把该“放”的放到位、该“管”的管到底、该“服务”的服务好。加快政务服务标准化、“互联网+政务服务”、远程异地评标试点建设，实行政务服务延时错时预约服务工作制，努力把宜春打造成全省营商环境最优市。

（五）锤炼本领，建设过硬型政府。注重加强学习，着力提高研判形势、把握全局、推进项目、驾驭风险、破解难题的能力。政府干的，应该是群众所盼的。要坚持科学、民主、依法决策，坚持依法全面履职，维护宪法和法律权威，加快推进法治宜春建设。自觉接受同级人大及其常委会的法律监督、工作监督，人民政协的民主监督，主动接受监察、司法、社会和舆论监督，让权力在阳光下运行。落实全面从严治党要求，坚决全面彻底肃清苏荣案余毒，持之以恒正风肃纪，严厉整治群众身边的腐败和作风问题，确保干部清正、政府清廉、政治清明。

各位代表，实干托起梦想，奋斗成就幸福。让我们更加紧密地团结在以习近平同志为核心的党中央周围，在省委、省政府和市委的坚强领导下，不忘初心、牢记使命，担当实干、感恩奋进，为把宜春建设成为区域中心城市、重塑宜春历史荣光不懈奋斗！
